# Deciphering the evolutionary affiliations among bacterial strains (*Pseudomonas* and *Frankia* sp.) inhabiting same ecological niche using virtual RFLP and simulation-based approaches

**DOI:** 10.1007/s13205-016-0488-5

**Published:** 2016-08-23

**Authors:** Meenakshi Srivastava, Manish Singh Kaushik, Amrita Srivastava, Anumeha Singh, Ekta Verma, Arun Kumar Mishra

**Affiliations:** Laboratory of Microbial Genetics, Department of Botany, Banaras Hindu University, Varanasi, 221005 India

**Keywords:** *Pseudomonas* sp., *Frankia* sp., 16S rRNA gene sequences, Virtual RFLP, Phylogeny

## Abstract

To decipher an evolutionary lineage between two different but important bacterial groups, i.e., *Pseudomonas* strain (γ-Proteobacteria) and *Frankia* strain (actinobacteria) growing in the same ecological niche in and around of an actinorhizal plant *Hippophae salicifolia* D. Don, genetic diversity and comparative molecular phylogeny have been investigated using 16S rRNA gene sequences and computer-simulated and virtually directed restriction fragment length polymorphism (RFLP) through 10 restriction enzymes. Bayesian and coalescent analyses on the basis of 16S rRNA gene sequences suggested three major groups with close proximity between *Pseudomonas* and *Frankia* isolates. This result has been further validated based on the data observed through similarity coefficient value and computational RFLP. Principal component analysis and Mandel h and k statistical analysis also confirmed and strengthen the findings. Approximately 458 aligned sequence of all the taxa were used to decipher nucleotide diversity, polymorphism and gene flow between these taxa. Thus, our results suggest for a possible co-evolution or a heterologous gene transfer of distantly related microbial forms. Further, our study also advocate for the use of computer aided, virtual RFLP analysis as a cost effective and rapid identification tool.

**Electronic supplementary material:**

The online version of this article (doi:10.1007/s13205-016-0488-5) contains supplementary material, which is available to authorized users.

## Introduction

Soil provides an enormous habitat for almost all kinds of microorganisms (Roger-Estrade et al. 2010). Diversity of microbial community and their resemblance to each other is very important factor in soil microhabitat and it is very crucial and mandatory to maintain soil health and other ecological interactions (Brock et al. 1994; Liesack and Stackebrandt 1992). Bacteria are found in all kinds of environment (Mulder et al. 2005), some groups of them occur more frequently and exhibit a very wide distribution, pseudomonads are one such example belongs to γ-proteobacteria group and are found free living or attached to the soil. These soil bacteria are ubiquitous in rhizosphere and possibly interact with the roots of plants and/or vice versa (Lynch and de Leij 1990). While *Frankia* strains either growing in free living condition or form symbiotic association with numbers of actinorhizal plants. (Sarma et al. 2006; Singh et al. 2008, 2009, 2010). It inhabits the root nodule of the non-leguminous, angiospermic *Hippophae salicifolia* D. Don, distributed along the high altitude areas of the Eastern Himalayas (North Sikkim) in India. Several other microbial communities might also be exists in the surrounding rhizosphere which definitely affects the soil properties of that place. A concerted approach regarding their phylogenetic affiliation must be needed to explore and analyze their diversity.

Characterization of *Pseudomonas* sp. has traditionally been done either through cultivation (Gould et al. 1985; Sugimoto et al. 1990), based on genetic study and biochemical tests (Anzai et al. 2000; Grimot et al. 1996), through molecular sequence analysis (Woese et al. 1984; Rajwar and Sahgal 2016) or FAME profiling (Vancanneyt et al. 1996). Some recent studies were also utilized RFLP gel electrophoresis and computer-simulated restriction analysis for the classification of fluorescent *Pseudomonas* sp. from cultured or uncultured strains (Gonzalez et al. 2000; Laguerre et al. 1994). Random genome fragments and DNA microarray technology were also used (Cho and Tiedje 2001). *Frankia* sp. have also been characterized using physiological, biochemical, molecular approaches (Normand et al. 1996; Singh et al. 2010) and RFLP-based analyses of 16S-ITS of *Frankia* (Khan et al. 2009).

Although a number of molecular tools have been used to elucidate the genetic diversity among the microorganisms but small subunit ribosomal RNA (SSU rRNA) sequences were found to be most commonly used molecular marker for phylogenetic as well as species level characterization due to its hypervariable as well as conserved regions which helps to resolve phylogeny and variability among strains more precisely (Clarridge 2004). 16S rRNA is widely use molecular marker for depict phylogeny among strain (Sen et al. 2015). RFLP data although, provides less direct information on the evolution of DNA sequences, but restriction sites because of their variability in sequences widely used in phylogeny and diversity studies and can be used directly to reveals informative sites or footprints of any organism. Restriction enzymes (type II) with 4 or 5 base cutter have been widely used to analyze restriction patterns (Pingoud and Jeltsch 2001). Restriction digestion generates discrete operational taxonomic units (OTUs) which represent different RFLP groups of interest and were used for exploratory data analysis. Virtual gel plotting or simulation-based analysis is further a step ahead in this process and have been previously employed to assess genetic variability among phytoplasma strains (Wei et al. 2007; Cai et al. 2008), as well as to develop a comprehensive classification scheme (Lee et al. 1998, 2000). Efficiency of different restriction enzymes for detecting and differentiating bacterial taxa (OTUs) on the basis of their representative SSU rRNA gene sequence have been reported by Moyer et al. (1996). RFLP gel electrophoresis and computer-simulated restriction analysis, for classification of fluorescent *Pseudomonas* sp. and bacteria have also been carried out by several workers (Gonzalez et al. 2000; Laguerre et al. 1994). Network analysis and sharing of antibiotic resistance genes between actinobacteria and γ-proteobacteria groups were analyzed previously (Tamminen et al. 2012). However, no work has been done till date that deals with the genetic diversity and evolutionary relationship among these two groups of bacteria growing in and around the root of *Hippophae salicifolia* D. Don. As they have shared the same ecological niche, some kind of genetic rearrangement might be possible among such microbial forms.

Therefore, the present investigation aimed to decipher the evolutionary relationship between the *Pseudomonas* (γ- proteobacteria) and *Frankia* (actinobacteria) strains using a concerted 16S rRNA gene sequence-based genetic diversity approach and bioinformatics or computer aided comparative RFLP analysis.

## Materials and methods

### Isolation and identification of bacterial strains

Isolation of rhizospheric *Pseudomonas* from surrounding actinorhizal plant was carried out by serial dilution and further culturing and sub culturing on agar plates. Strains were cultivated at 30 °C in King’s B medium (King et al. 1954) adjusted to a pH of 7.2. Among different bacterial isolates, four were selected for a detailed study. The selection was based on their colony morphology, growth characteristics and other biochemical properties such as pH and temperature tolerance of isolates on agar medium. *Frankia* strains isolated from nodules of *Hippophae salicifolia* D. Don (Seabuckthorn) were cultured and maintained according to standard protocol (Murry et al. 1984).

### 16S rRNA-PCR amplification

16S rRNA amplification for 16S rRNA gene was done using primers fD1(5′-AGAGTTTGATCCTGGCTCAG-3′) and rD1 (5′-AAGGAGGTGATCCAGCC-3′) (Weiseburg et al. 1991). Each PCR reaction was performed in 25 μl aliquots containing 10–20 ng DNA template, 10.0 μM of each primer, 1.5 mM MgCl_2_, 200 μM dNTPs, and 1 U/μl Taq polymerase. The template was initially denatured at 95 °C for 5 min. This was followed by 32 cycles of denaturation for 15 s at 95 °C, 30 s of annealing at 54 °C and 1 min of extension at 72 °C, followed at last by the final extension step of 5 min at 72 °C. After running in 1.2 % agarose gels, the amplified products were visualized on Bio Rad Gel documentation system.

### Nucleotide sequence accession numbers

The nucleic acid sequences of *Pseudomonas* species and *Frankia* strains used in the present study have been deposited to NCBI database using the submission tool Sequin and for the same accession numbers were obtained. Other sequences used in the computational study were retrieved from NCBI and listed along with isolated strains (Table 1).

**Table 1:** Strains selected in study

RFLP groups	Selected taxa	Division	Strain designation	Accession number
a	*Frankia* sp.	Actinobacteria	G2	JN685209
b	*Frankia* sp.	Actinobacteria	–	L40622
c	*Frankia* sp.	Actinobacteria	FE12	AF158687
d	*Frankia* sp.	Actinobacteria	BMG5.11	AM040443
e	Uncultured *Acidovorax* sp.	β-Proteobacteria	Clone SB38	JQ723680
f	Uncultured *Comamonas* sp.	β-Proteobacteria	Clone DS091	DQ234174
g	Uncultured bacterium	–	Clone marine heat A5	HM363289
h	*Pseudomonas* sp.	γ-Proteobacteria	AMD3	EU600210
i	*Pseudomonas* * stutzeri*	γ-Proteobacteria	TH-31	KF783212
j	Uncultured *Pseudomonas* sp.	γ-Proteobacteria	–	KF733608
k	*Pseudomonas* *stutzeri *	γ-Proteobacteria	ATCC 17588	AF094748
l	*Pseudomonas* sp.	γ-Proteobacteria	IND 1	KJ911224
m	*Pseudomonas* sp.	γ-Proteobacteria	IND 2	KJ911225
n	*Pseudomonas* sp.	γ-Proteobacteria	IND3	KJ911226
o	*Pseudomonas* sp.	γ-Proteobacteria	IND 4	KJ911227
p	*Frankia* sp.	Actinobacteria	HsIi2	JQ480013
q	*Frankia* sp.	Actinobacteria	HsIi8	JQ480011
r	*Frankia* sp.	Actinobacteria	HsIi9	JQ480009
s	*Frankia* sp.	Actinobacteria	HsIi10	JQ480012

### Retrieval of 16S rRNA gene sequences and Bayesian phylogenetic analyses

For clear picture of cladistic analysis, reference strains of different proteobacteria and some *Frankia* strain which were more closely related from sequence data were retrieved from NCBI’s nucleotide sequence database (http://www.ncbi.nlm.nih.gov/gquery/gquery.fcgi) using the Entrez search and retrieval tool (Wheeler et al. 2005). All sequences were subjected to BLAST search (www.ncbi.nlm.nih.gov/blast) and thus the closest relatives obtained from GenBank were included in the subsequent phylogenetic reconstructions and population analysis. Multiple sequence alignment was performed using the ClustalW tool within alignment function of MEGA 5.2 phylogenetic package (Tamura et al. 2011). Bayesian analysis of sequence data were performed using BEAST v1.6.1 (Drummond and Rambaut 2007; Drummond et al. 2012). An uncorrelated lognormal distribution in clock estimation (Drummond et al. 2006) and a tree prior were used with a coalescent process. An independent runs for 100 millions of generations was used with sampling every 1000 generations. Tracer v1.5 was used to examine convergence (Rambaut and Drummond 2009). Results were obtained and visualized in the Tree Annotator and Fig tree software (Rambaut and Drummond 2009).

Sequence-based phylogenetic trees were computed and resolved using the four format of tree construction, i.e., minimum evolution (ME), maximum parsimony (MP), maximum likelihood (ML), and neighbor joining (NJ) algorithms and bootstrap support value for all methodology were shown (Fig. S1). To infer evolutionary cladistic analysis, nucleotide positions containing gaps and missing data were eliminated from the data set through complete deletion option. The robustness of the internal branches of the trees were estimated by bootstrap analyses using 1000 replications with bootstrap majority rule (>50 %) in a heuristic search (Vinnere et al. 2002). Finally consensus tree was obtained (data are not given).

The phylogenetic tree based on the bioinformatics-based RFLP was constructed using NTSYS 2.02 version (Rohlf 1998). To analyze genetic diversity and gene flow among selected strains, their groups were assigned. The number of segregating sites (*s*), parsimony informative sites, nucleotide diversity per site (*P*_i_) based on the average number of pair-wise differences per site (*k*) was calculated within and between them (Nei 1987). Recombination was also determined for all groups. Program DnaSP v. 5.1 was used to estimate all these parameters (Librado and Rozas 2005).

### In silico restriction enzyme digestions

All sequences selected for study were aligned together, trimmed and checked individually for both phylogenetic as well as simulatory RFLP extrapolatory data analysis. As the length of these retrieved sequences ranges between few hundred bases to full-length rRNA, multiple alignment of sequences were carried out to compare sequence data, aligned in Bioedit using ClustalW (Thompson et al. 1994) and compared with each other to get sequence homology as well as differences. Only aligned regions were subjected to further downstream experiments. Screening of DNA sequence data for variable endonuclease restriction sites among representative species were carried out by NEB cutter, version 2.0, available via a web server (http://tools.neb.com/NEBcutter) that accept an input DNA sequence and produce a comprehensive report of the restriction enzymes for the target sequences (Vincze et al. 2003). This aligned and trimmed sequences were then subjected to in silico restriction analysis and virtual gel plotting. Fragments were digested in silico with 10 distinct restriction enzymes that have been routinely used for different 16S rRNA gene RFLP analysis (Lee et al. 1998). These enzymes were *AluI* (AG’CT)*, BstUI* (CG’CG), *DdeI* (C’TNAG), *HaeIII* (GG’CC), *HhaI* (GCG’C), *HinfI* (G’ANTC), *MboI* (‘GATC), *MspI* (C’CGG), *RsaI* (GT’AC), *and TaqI* (T’CGA). After restriction digestion, a virtual gel image was created automatically (Data are not given), that used for further RFLP pattern comparisons and statistical as well as in silico analysis. A similarity coefficient (*S*_j_) was calculated for each pair of selected strains according to the formula of Jaccard similarity coefficient for binary data (Everitt et al. 2001) that formed a contingency table for any two objects *i* and *j* of a set, on the basis of formula,

$$S_{j} = \, a/a + b + c$$

where *a* number of variables on which both objects *i* and *j* are 1.

*b* number of variables where object *i* is 1 and *j* is 0.

*c* number of variables where object *i* is 0 and *j* is 1.

### Statistical simulation of molecular data

Statistical validation of the outcomes was performed using principal component (PCA) analysis by Biodiversity pro software (ver. 2). Data obtained in terms of coordinates were exported to Sigma plot 11 and used to generate the graphical representation of the values.

Mandel’s *h* and *k* statistics was carried out through software (XLSTAT-Pro 7.5 Addinsoft, New York, USA). Statistics for replicate observations was done, assuming that the observations are identically distributed and follow a normal distribution. We calculated Mandel’s *h*_i_ for group *i* (*i* = 1…p) given by.

$$h_{\text{i }} = \frac{{\overline{x} - \overline{\overline{x}} }}{s}$$

Mandel’s *h* is an indication of relative deviation from the mean value. Critical values and confidence intervals for a given level of significance α around statistic h can be calculated (Wilrich 2013).

$$h_{\text{crit }} \left( {p,\, a} \right)\; = \;\frac{{\left( {p - 1} \right)t_{y - 2,1\, - \,a/2} }}{{\sqrt {\left( {p(p - 2 + t_{y - 2,\,1 - a/2}^{2} } \right)} }}$$

Mandel’s *k* is an indicator of precision compared to the pooled standard deviation across all RFLP groups. Mandel’s (*k*_i_) for group *i* (*i* = 1…p) is given by:

$$k_{\text{i}} = \frac{{s_{\text{i}} }}{{\widetilde{s}}}$$

with

$$s_{\text{i}} = \frac{1}{{n_{\text{i}} - 1}}\mathop \sum \limits_{j = 1}^{{n_{\text{i}} }} \left( {x_{ij} - \overline{{x_{i} }} } \right)^{2} {\text{and}}\;\overline{s} = \sqrt {\frac{1}{p}\mathop \sum \limits_{i = 1}^{p} s_{i}^{2} }$$

where the critical value is given by:

$$k_{\text{crit }} \left( {n, \,a} \right) = \;\sqrt {p(1 + \left( {p - 1} \right)F_{{1 - a,\;\left( {y - 1} \right)\left( {x - 1} \right)\left( {x - 1} \right)}}^{ - 1} } )$$

## Results

Two separate phylogenetic approaches (sequence and RFLP-based) were performed in order to establish clear criteria for grouping two different groups of rhizospheric bacteria. The phylogeny reconstruction using four different methods: minimum evolution (ME), maximum likelihood (ML), maximum parsimony (MP) and neighbor joining (NJ) were analyzed and all methods gave a similar clustering appearance in the Bayesian and typical phylogenetic analyses.

### Phylogenetic analysis

**Fig. 1:**
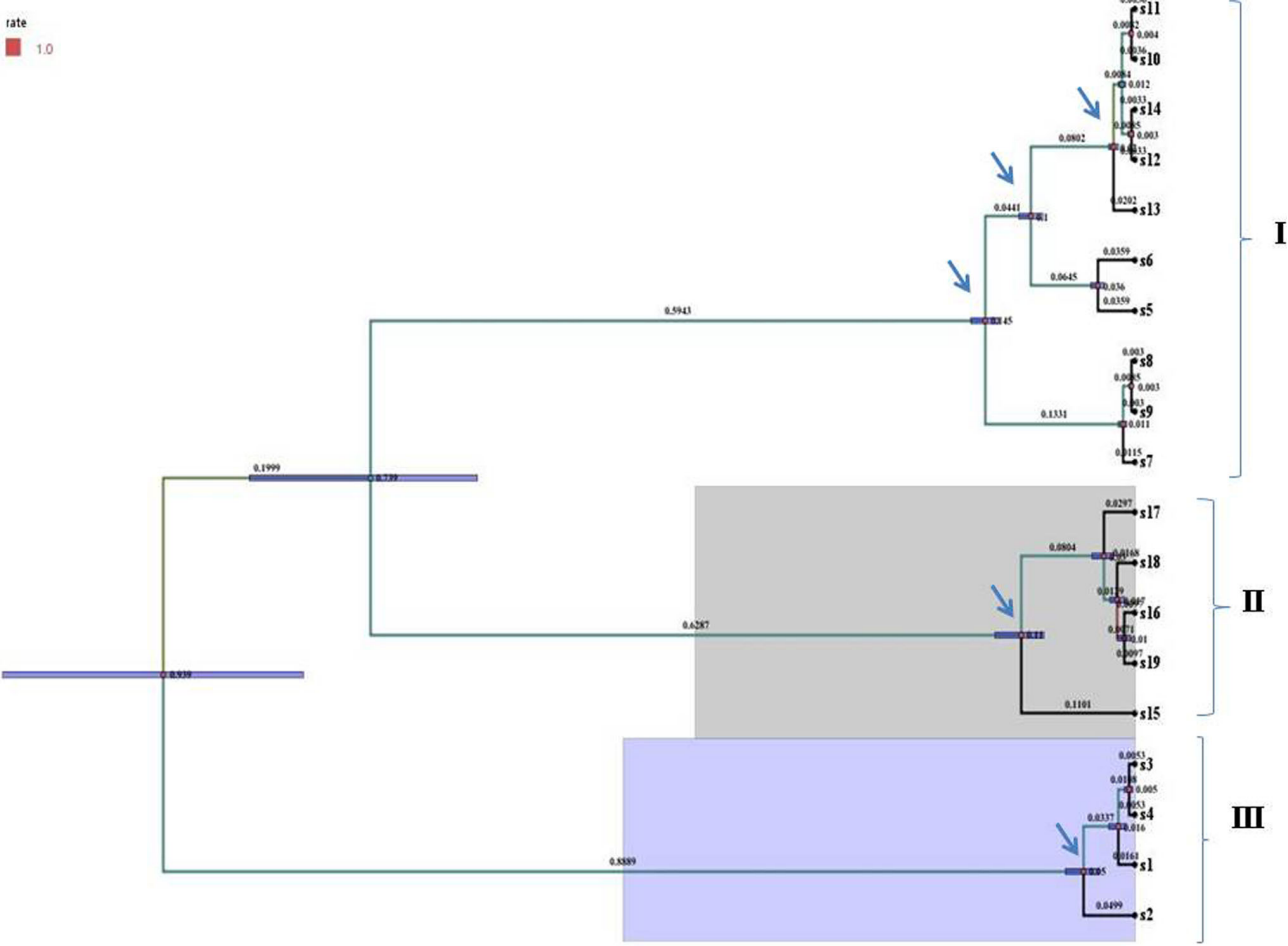
The evolutionary relation between strains was inferred using the Bayesian phylogenetic tree. 1,000,000 generations for BI (Bayesian inferences) using the GTR+ G model was done. Branches corresponding to partitions reproduced in less than 50 % bootstrap replicates are collapsed. Node bars and branch length were given, arrow represents selected nodes for bootstrap support value estimation. *Pseudomonas* sp. IND1 (S1), *Pseudomonas* sp. IND2 (S2), *Pseudomonas* sp. IND3 (S3), *Pseudomonas* sp. IND4 (S4), Uncultured *Acidovorax* sp. clone (S5), Uncultured *Comamonas* sp. (S6), *Frankia* sp. G2 (S7), *Frankia* sp. (S8), *Frankia* sp. FE12 (S9), Uncultured bacterial sp. (S10), *Pseudomonas* sp. AMD3 (S11), *Pseudomonas stutzeri* strain (S12), Uncultured *Pseudomonas* sp. (S13), *Pseudomonas stutzeri* ATCC (S14), *Frankia* sp. BMG5.11 (S15), *Frankia* sp. Hsli 2 (S16), *Frankia* sp. Hsli 8 (S17), *Frankia* sp. Hsli 9 (S18), *Frankia* sp. Hsli 10 (S19)

Total nineteen sequences of different bacterial isolates have been investigated in the present study (Table 1). The evolutionary history was inferred using Bayesian phylogeny method (Fig. 1). The bootstrap consensus tree inferred from 1000 replicates was taken to represent the evolutionary history of the taxa analyzed. The evolutionary distances were computed using the p-distance method and are in the units of the number of base differences per site. There were a total of 100 positions in the final dataset and all positions containing gaps and missing data were eliminated and evolutionary analyses were conducted in MEGA5 (data are not given). As this is a study of two divergent groups so Bayesian phylogenetic analysis was used. Tree topology and clustering behavior were found to be similar from both the analysis and a unique tree was presented out of millions trees with the bootstrap values and posterior probabilities greater than 50 % and 0.95, respectively.

The tree revealed three major clusters (one large cluster with three sub clusters), comprised of mix assemblage of strains with uncultured bacterium (HM363289), *Pseudomonas* sp. (EU600210), *Pseudomonas stutzeri* (KF783212) *Pseudomonas stutzeri* (AF094748) and uncultured *Pseudomonas* (KF733608) in one subclade where uncultured *Acidovorax* sp. as well as uncultured *Comamonas* sp. form another group. Others reference actinobacteria merged into third subgroup. Isolated *Pseudomonas* sp., i.e., *Pseudomonas* IND1, *Pseudomonas* sp. IND2, *Pseudomonas* sp. IND3 and *Pseudomonas* sp. IND4 merged into a separate cluster (III). With very close vicinity of cluster of *Pseudomonas* isolates, Cluster II comprised *Frankia* sp. Hsli2, *Frankia* sp. Hsli8, *Frankia* sp. Hsli9 and *Frankia* sp. Hsli10 isolated from same ecological niche of root nodules of *Hippophae salicifolia* D. Don.

### Simulated RFLP analysis

**Fig. 2:**
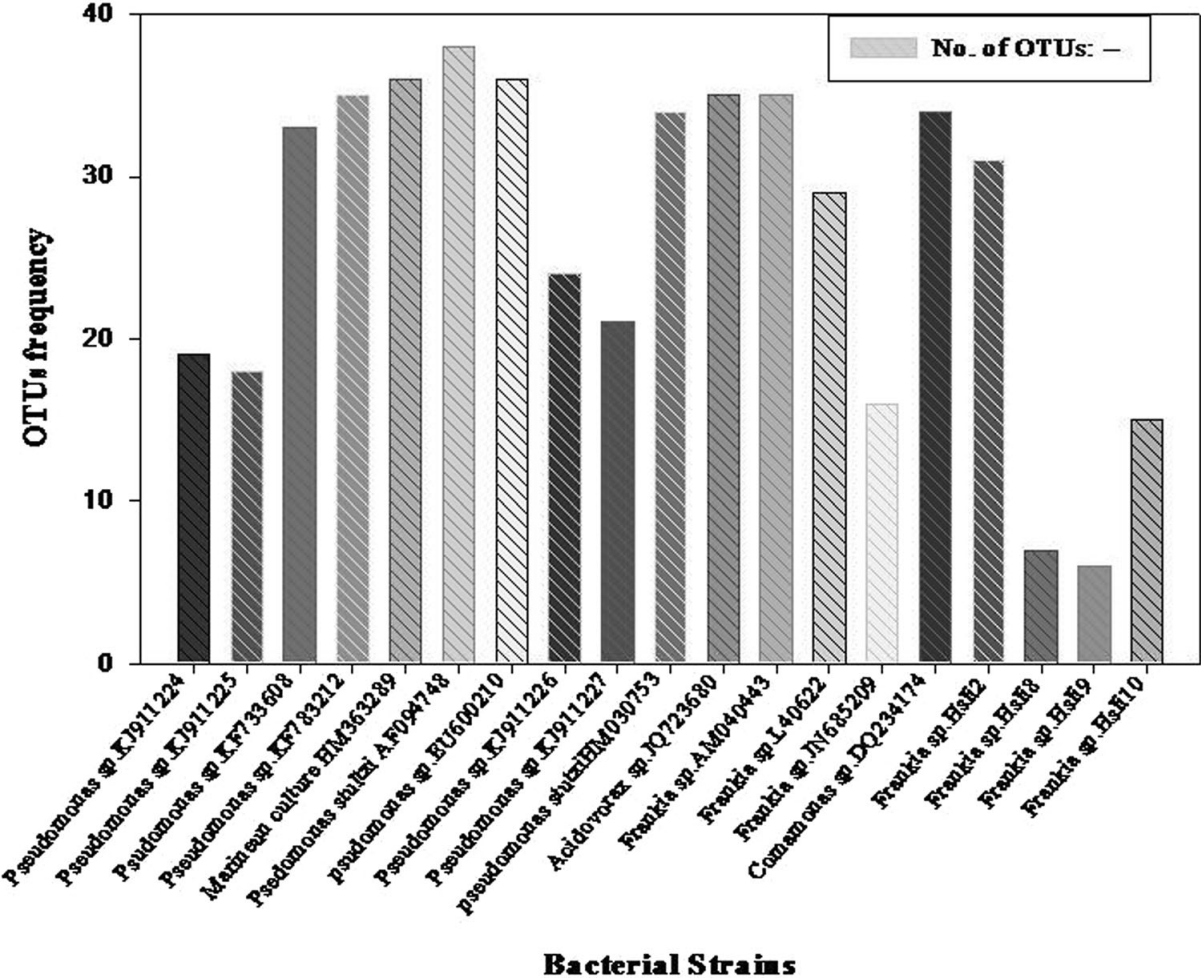
OTUs generated through different RFLP groups

**Fig. 3:**
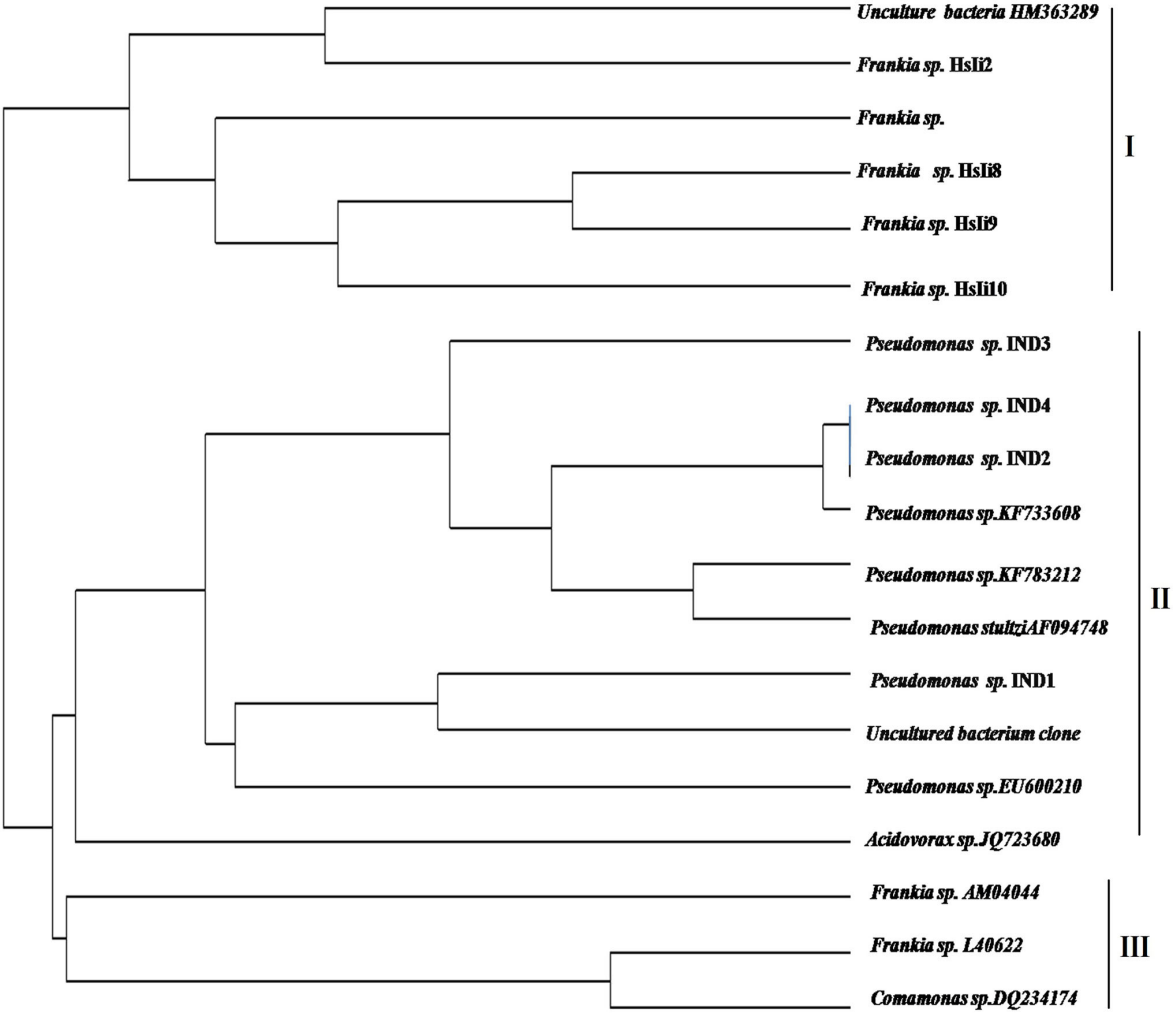
Dendrogram of selected strains based on computer aided RFLP and generated using the NTSYS-pc, version 2.00 (Rohlf 1998) program

Based on occurrence of restriction sites for each and every restriction endonuclease enzyme, strains were distributed into different RFLP groups (Table 2). RFLP maps were generated through NEBcutter, version 2.0 for isolated *Pseudomonas* strains (Fig. S2) and similarly generated for all strains too (Data are not given). OTUs frequency generated after restriction digestion of different RFLP groups were shown in Fig. 2. UPGMA dendrogram was generated using jaccard similarity coefficient for binary data, shown three different cluster combinations among isolates (Fig. 3). Cluster I comprised of uncultured bacterial clone, *Frankia* sp. Hsli2 with *Frankia* sp. Hsli8, *Frankia* sp. Hsli9, *Frankia* sp. Hsli10. Cluster II consisted of 10 isolates, *Pseudomonas* sp. IND4 and *Pseudomonas* sp. IND3 showing very close mergence with another isolated *Pseudomonas* sp. with reference *Pseudomonas* strain and uncultured *Comamonas* sp. Cluster III comprised of *Acidovorax* and *Comamona*s strain and two *Frankia* sp. The similarity score calculated by Jaccard coefficient used to construct UPGMA (unweighted pair-group method with arithmetic averages, also known as average linkage) dendrogram from the binary data were estimated (Table S1).

**Table 2:** Restriction map of various RFLP groups

REs	a	b	c	d	e	f	g	h	i	j	k	l	m	n	o	p	q	r	s
*AluI*	+	+	+	+	+	+	+	+	+	+	+	+	+	+	+	−	−	+	+
*BstUI*	+	+	+	+	+	+	+	+	+	+	+	+	+	+	+	+	−	+	+
*DdeI*	+	−	+	+	−	+	+	+	+	+	+	+	+	+	+	−	+	+	+
*HaeIII*	+	+	+	+	+	+	+	+	+	+	+	+	+	+	+	+	+	+	+
*HhaI*	+	+	+	+	+	+	+	+	+	+	+	+	+	+	+	+	+	+	+
*HinfI*	+	+	+	+	+	+	+	+	+	+	+	+	+	+	+	−	−	−	−
*MboI*	+	+	+	+	+	+	+	+	+	+	+	+	+	−	+	+	−	+	+
*MspI*	+	+	+	+	+	+	+	+	+	+	+	−	+	+	+	−	+	+	+
*RsaI*	+	+	+	+	+	+	+	+	+	+	+	+	+	+	+	+	+	+	+
*TaqI*	+	+	+	+	+	+	+	+	+	+	+	+	+	+	+	−	−	+	+

(+) denotes the presence of the site and (−) absence of the site

### Principal component analysis

**Fig. 4:**
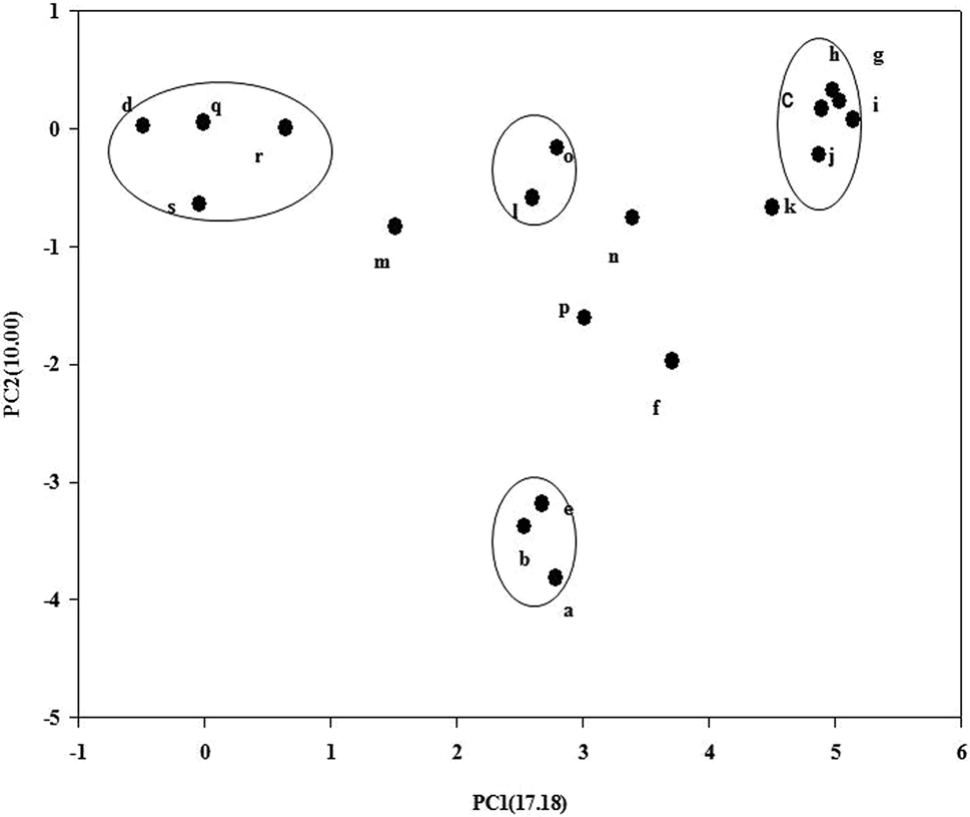
PCA analysis of RFLP patterns: uncultured *Pseudomonas* sp. (j) *Frankia* sp. FE12 (c) Uncultured bacterial sp. (g) *Pseudomonas* sp. IND3 (n) *Pseudomonas* sp. IND1 (l) uncultured *Acidovorax* sp. clone (e) *Pseudomonas stutzeri* strain TH-31 (i) *Pseudomonas* sp. IND2 (m) *Pseudomonas* *stutzeri* ATCC 17588 (k) *Pseudomonas* sp. AMD3 (h) *Pseudomonas* sp. IND4 (o) *Frankia* sp. G2 (a) Uncultured *Comamonas *sp. (f) *Frankia* sp. (b) *Frankia* sp. BMG5.11 (d) *Frankia* sp. Hsli 2 (p) *Frankia* sp. Hsli 8 (q) *Frankia *sp. Hsli 9 (r) *Frankia* sp. Hsli 10 (s)

PCA analysis was performed and showed the similar kind of pattern and reveals four major clusters (Fig. 4). *Frankia* sp. G2, uncultured *Comamonas* sp. clone, *Frankia* sp. and *Pseudomonas* sp. KJ11227 form one large assemblage, *Pseudomonas* *stutzeri* TH31, *Acidovorax* sp. clone acquired one cluster and *Pseudomonas* sp. IND4 and *Pseudomonas* sp. IND1 form one group and *Pseudomonas* sp. IND2, *Pseudomonas* sp. IND3 and *Frankia* sp. Hsli 2 form a separate grouping. While *Frankia* sp. Hsli 8, *Frankia* sp. Hsli 9, *Frankia* sp. Hsli 10 form another separate cluster in principal component analysis.

### Statistical analysis and genetic diversity study

Mandel’s h and k statistics have been used to check the consistency of the data. “Mandel’s h” provides the inter-laboratory consistency statistic and the “Mandel’s k” define intra-laboratory consistency stat, mainly graphical extrapolation of tested strain in terms of errors and deviation was evaluated through this parameter (Fig. S3). To identify groups, for which the variance is potentially abnormal, critical values and confidence intervals for a given level of significance a around statistic h and k were determined. *Pseudomonas* sp. IND1, *Pseudomonas* sp. IND2* Pseudomonas* sp. IND3, *Pseudomonas* sp. IND4 exhibit more or less same kind of graphical exploratory pattern with *Frankia* sp. L40622 as well as resemblance to *Frankia* strain Hsli10, where deviation was found to be in range of 0.19–0.25.

Out of 19 RFLP groups, only aligned 458 bps were selected to study DNA polymorphism and nucleotide diversity within and between groups, data were compared by dividing different sequence sets into three groups according to their clustering behavior. Groups defined as isolated *Pseudomonas* sp., reference proteobacteria and actinobacteria, respectively.

In total sequence sets 4 invariable or monomorphic sites, 86 variable or polymorphic sites with 160 total no. of mutations and 10 Singleton variable sites were found. In which total number of Haplotypes (h) are 14 and Parsimony informative sites are 76. Haplotype (gene) diversity (Hd) was 0.965 and total Nucleotide diversity (Pi) was 0.46407 comprising average number of nucleotide differences (k), 41.766. Cumulative diversity between groups was also determined. Average number of nucleotide differences (k) and total number of mutations were found to be 135.964 and 423 for *Pseudomonas* and others proteobacterial group and 58.781, 120 between *Pseudomonas* and actinobacterial group (Table S2).

## Discussion

The phylogenetic affiliation between two groups of bacterial strains growing together has not been disputed as such and previous studies only adressed the influence of *Pseudomonas* and other bacterial strains on *Frankia* nodulation (Knowlton and Dawson 1983). *Frankia* sp. could act as associative nitrogen fixers with some host as well as non host plants have already been described (Singh et al. 2010), but a clear cut phylogenetic association of these two divergent bacterial groups have not been well understood. So, this is the first kind of study to infer phylogeny using combination of wet lab and dry lab approaches. Where, phylogenetic analyses were carried out on the basis of Bayesian phylogeny of 16S rRNA gene sequences and computer-based RFLP coupled with statistical as well as some population genetics study. In this paper, the simulation-based or computational RFLP pattern analysis was carried out through restriction digestion of 16S rDNA gene fragments which was further used to generate RFLP data equivalent to ribotyping and provided a better means of describing a bacterial community and its diversity in soil for this study but should be equally applied for other habitats. Based on logistic simplicity for analysis and occurrence of restriction sites in different strains, ten different TREs were selected for virtual restriction digestion, which divided each RFLP group into a number of operational taxonomic units for further extrapolation.

Members of two different bacterial groups were showed mixed grouping pattern throughout the tree constructed. Bayesian tree suggested a clear cut differentiation among the strains with little intermixing of some proteobacterial as well as actinobacterial forms in clade I, whereas isolated *Frankia* sp. and *Pseudomonas* IND sp. formed separate cluster (II, III). Uncultured *Acidovorax* sp as well as uncultured *Comamonas* sp. of group β-proteobacteria merge into a separate sub-group that also satisfies the morphological criteria of their classification (Woese 1987). Interestingly, there were complete divergence of isolated *Pseudomonas* sp., i.e., *Pseudomonas* IND1, *Pseudomonas* sp. IND2, *Pseudomonas* sp. IND3 as well as *Pseudomonas* sp. IND4 which formed a separate clade (III). A single tree is presented with bootstrap values and posterior probabilities greater than 70 % and 0.95, respectively. For these two bacterial groups, phylogenetic connection are still not available but analyses of network metrics and sharing of antibiotic resistance genes between actinobacteria and γ-proteobacteria groups were analyzed previously (Tamminen et al. 2012). To analyze RFLP-based phylogeny, maps were generated and thus obtained restriction fragments were considered as OTUs pattern and further used for cladistic analysis. Although not all but the major portion of the phylogenetic reconstruction deduced were in sync with the tree obtained by the sequence analysis. UPGMA dendrogram was generated using Jaccard similarity coefficient for binary data, shown three different cluster combinations among isolates, where I clade consisted of most of the* Frankia* strains with one uncultured bacterial clone and II cluster comprised of *Pseudomonas* spp. This observation was dependent on restriction digestion of aligned 16S rRNA region of all strains. The similarity score calculated by Jaccard coefficient used to construct UPGMA (unweighted pair-group method with arithmetic averages, also known as average linkage) dendrogram from the binary data were estimated and analyses by Jukes-Cantor model that showed a wide range of proximity distribution among strains and range between 0.040 and 0.821.

An interesting observation was noticed by the RFLP-based phylogenetic tree. Coherence between UPGMA as well as Bayesian phylogenetic tree placed our lab isolates in close proximity of each others rather than their respective reference strains suggested that there might be an existence of a possible phylogenetic linkage/connection that need to be resolve between such kind of distantly related microorganisms. This finding is supported with the observations made by Tamminen et al. (2012). Thus, results obtained showed immense diversity and differences in the small subunit ribosomal RNA (SSU rRNA) genes sequences and the bioinformatics-based RFLP along with population genetics, confirm our findings and substantiate our data prior to reporting anything based only on the phylogenetic tree. Virtual gel plotting or simulation based analysis were assessed previously in four-enzyme-based MERFLP screening protocol to distinguish *Pseudomonas* environmental libraries (Porteous et al. 2002) as well as for Phytoplasma strains (Wei et al. 2007; Hong Cai et al. 2008; Lee et al. 2000).

Further, extrapolation of DNA sequence data into mathematical form helps to evaluate the evolutionary pace in mostly selective group of organisms (Srivastava et al. 2015, 2016). So, to derive genetic diversity within and between the groups, population structure were analyzed, showed recombination frequency R per gene ranges from 0.5 (*Pseudomonas* strains) to 0.001 (γ-Proteobacteria) and 2.7 (actinobacteria). Average number of nucleotide differences (k) and total number of mutations were also calculated and found to be more between *Pseudomonas *and proteobacteria group (135.964, 423) than *Pseudomonas* and actinobacterial strains (58.781, 120). Nucleotide diversity Pi(t) was found to be 0.43163 and 0.45207, respectively, shown minor differences between these two groups. On taking two closely related group of *Pseudomonas*, i.e., isolated strains and all proteobacterial reference strains, we found huge variations in the cut sites, restriction enzymes used to generate cuts as well as in different phylogenetic tree constructed. Further, results obtained through population structure analysis also support the findings. Consequently, statistical analysis such as PCA and Mandel’s h and k stat were performed, where PCA analysis were given a dimensions to each variable in form of coordinate and thus reflecting associative behavior of all variables (RFLP groups in present study). PCA analysis validated the possible assemblage of all RFLP groups according to their prior phylogenetic analysis based on tree construction as well as Bayesian study. Clustering of the entire representative taxa depicted a similar kind of association that can be withdrawn from traditional as well as RFLP-based phylogeny. Mandel h and k stat, as a test of outliers group, provides a rapid graphical view of intra-laboratory bias and relative precision. Thus, Coefficients of pattern similarity based on restriction fragment data as well as all other observations clearly established an evolutionary lineage between *Pseudomonas* strains with that of *Frankia* isolates growing in same habitat/ecological niche.

It is also very much evident that with respect to altered environment or habitat, changes occur in each and every phylotype and to validate phylogenetic relationship between microorganisms present in particular habitat/ecological niche, it is laborious and troublesome to done restriction analysis of each and every taxon with single restriction enzyme every time. So, cumulative digestion of taxon by different restriction enzymes using bioinformatics assisted method could be correlated or some time surpass the lab-based restriction digestion experiments to give greater resolution and a better insight about species identification as well as would be helpful for phylogenetic reconstruction analysis. In this study emphasis was laid on Bayesian-based phylogenetic approaches that revolutionize tree estimation in general through useful algorithms viz. MCMC-based algorithm. Evolutionary tree construction is now considered as a standard part of exploratory sequence analysis in all kind of phylogenetic studies. In this connection, Bayesian methods for estimating phylogenetic trees have been proposed as a faster method of incorporating the power of complex statistical models into the process. Such comparative analyses provide the theoretical and practical modulation that could not be determined through the traditional methods only. Simply it can be stated that the ability of the new approaches to address previously uncategorized questions through traditional means making computer-based or phylogenetic analysis an interactive tool in a wide areas of research. Cost effectiveness, lack of experimental biasness as well as generation of reproducible results make it a valuable and faster tool for phylogenetic assessment. Based on the above observations we conclude that (1) both, sequence-based phylogeny along with the new computer-based RFLP analyses is reliable and proven to be more reliable approaches for detecting strains affiliation, (2) multiple restriction digestion to detect OTUs through a PCR–RFLP analysis can gives a better picture of strains affiliation, (3) bioinformatics-based RFLP analysis suggests that all the strains comprised distinct, unique and specific band profile and high genotypic variability even between strains of the same group and (4) all parameters strengthen the findings and proven to be a baseline on which further study related to the evolutionary lineage or affiliation among different bacterial groups of similar ecological niche could be performed.

## Electronic supplementary material

Below is the link to the electronic supplementary material.

Fig. S1. Bootstrap support value for different phylogenetic reconstruction methods. Minimum evolution (ME), maximum likelihood (ML), maximum parsimony (MP) and neighbor joining (NJ). (TIFF 2067 kb)

Fig. S2. Restriction maps of four isolated *Pseudomonas* type strains through ten different restriction enzymes. *Pseudomonas* sp. IND1, *Pseudomonas* sp. IND2, *Pseudomonas* sp. IND3, *Pseudomonas* sp. IND4. (TIFF 1888 kb)

Fig. S3. Mandel h and k statistical analysis with significance level. Critical values (h Crit, k Crit) and confidence intervals for a given level of significance a around statistic h and k is given (TIFF 2273 kb)

Supplementary material 4 (DOCX 31 kb)

Supplementary material 5 (DOCX 23 kb)
