# Supplementary figures and images for: Deciphering the evolutionary affiliations among bacterial strains (Pseudomonas and Frankia sp.) inhabiting same ecological niche using virtual RFLP and simulation-based approaches
Source: 3 Biotech. 2016 Aug 23;6(2):178. doi: 10.1007/s13205-016-0488-5 (PMC4993716; doi:10.1007/s13205-016-0488-5)

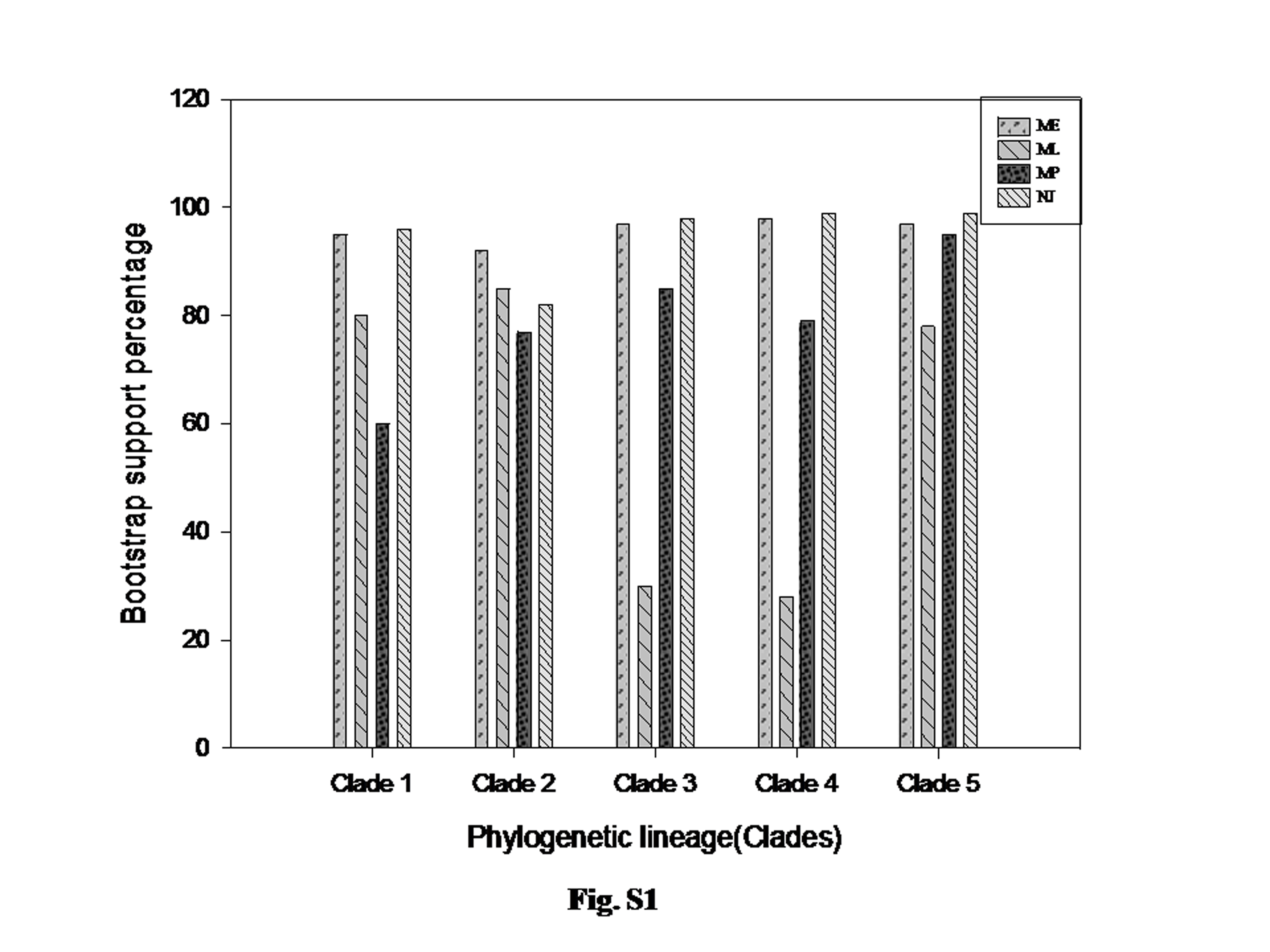

Supplement: Supplementary file 1 — Fig. S1. Bootstrap support value for different phylogenetic reconstruction methods. Minimum evolution (ME), maximum likelihood (ML), maximum parsimony (MP) and neighbor joining (NJ). (TIFF 2067 kb) [file 13205_2016_488_MOESM1_ESM.tif]

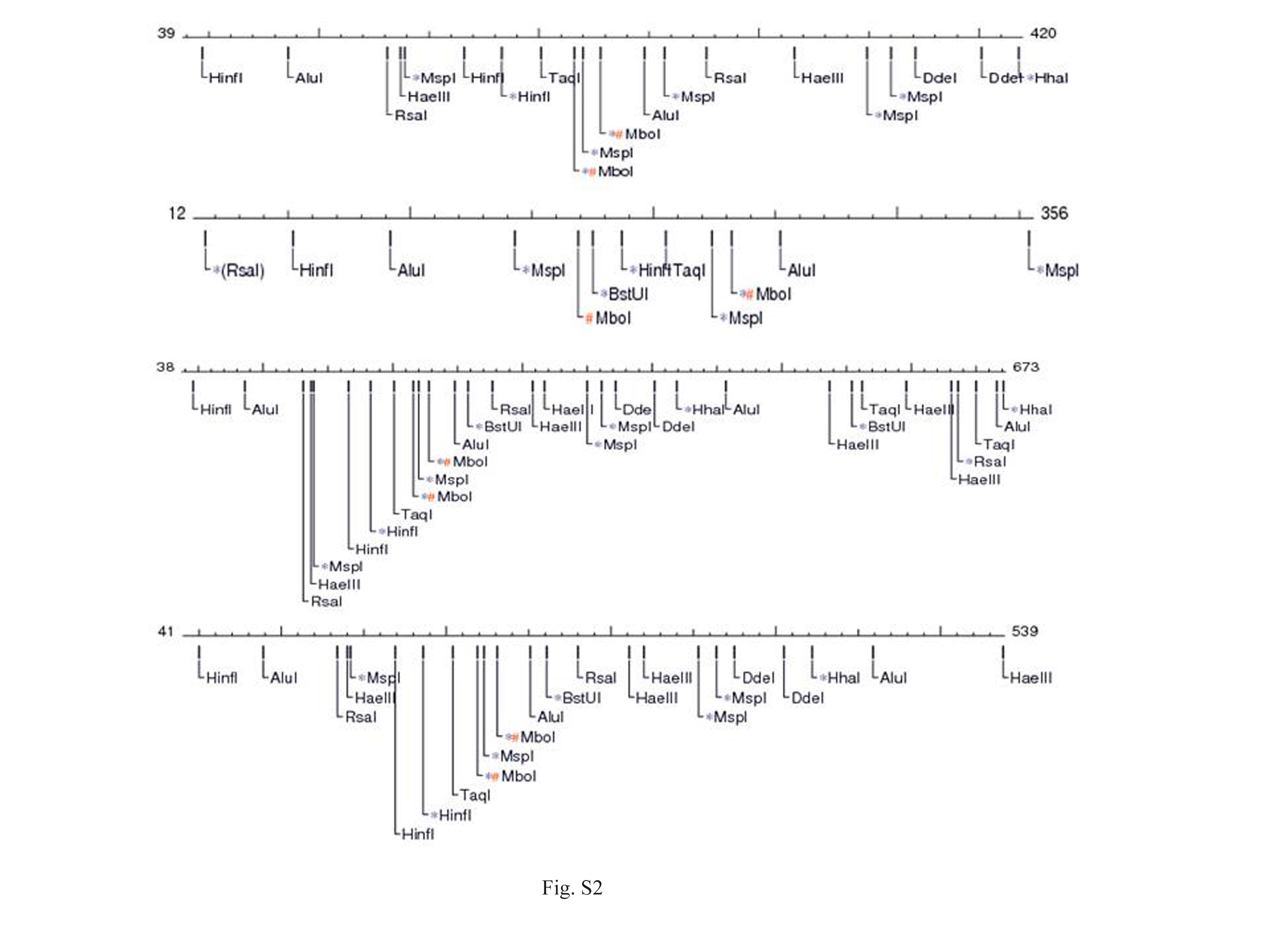

Supplement: Supplementary file 2 — Fig. S2. Restriction maps of four isolated Pseudomonas type strains through ten different restriction enzymes. Pseudomonas sp. IND1, Pseudomonas sp. IND2, Pseudomonas sp. IND3, Pseudomonas sp. IND4. (TIFF 1888 kb) [file 13205_2016_488_MOESM2_ESM.tif]

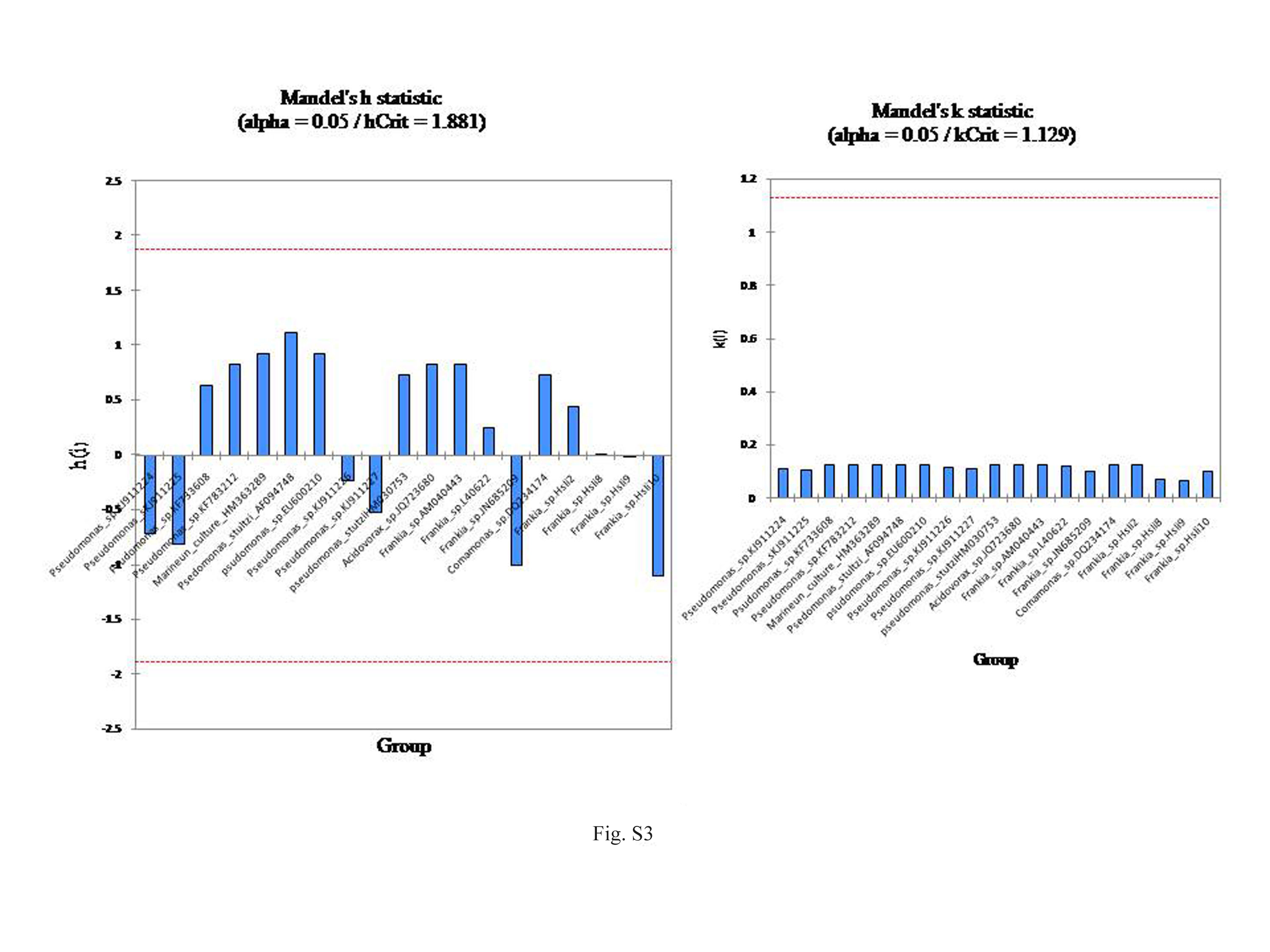

Supplement: Supplementary file 3 — Fig. S3. Mandel h and k statistical analysis with significance level. Critical values (h Crit, k Crit) and confidence intervals for a given level of significance a around statistic h and k is given (TIFF 2273 kb) [file 13205_2016_488_MOESM3_ESM.tif]
