# Supplementary material for: Deciphering the evolutionary affiliations among bacterial strains (Pseudomonas and Frankia sp.) inhabiting same ecological niche using virtual RFLP and simulation-based approaches
Source: 3 Biotech. 2016 Aug 23;6(2):178. doi: 10.1007/s13205-016-0488-5 (PMC4993716; doi:10.1007/s13205-016-0488-5)
Supplement: Supplementary file 4 — Supplementary material 4 (DOCX 31 kb) [file 13205_2016_488_MOESM4_ESM.docx]

|  | | | |  |  |  |  |  |  |  |  |  |  |  |  |  |  |  |  |
| --- | --- | --- | --- | --- | --- | --- | --- | --- | --- | --- | --- | --- | --- | --- | --- | --- | --- | --- | --- |
|  |  |  |  |  |  |  |  |  |  |  |  |  |  |  |  |  |  |  |  |
|  | (S1) | (S2) | (S3) | (S4) | (S5) | (S6) | (S7) | (S8) | (S9) | (S10) | (S11) | (S12) | (S13) | (S14) | (S15) | (S16) | (S17) | (S18) | (S19) |
| (S1) *Pseudomonas sp*.KJ911224 | 1 | 0.370 | 0.368 | 0.421 | 0.375 | 0.390 | 0.410 | 0.387 | 0.379 | 0.432 | 0.256 | 0.317 | 0.333 | 0.029 | 0.395 | 0.282 | 0.040 | 0.087 | 0.097 |
| (S2) *Pseudomonas sp*.KJ911225 | 0.370 | 1 | 0.308 | 0.293 | 0.256 | 0.302 | 0.256 | 0.273 | 0.258 | 0.300 | 0.262 | 0.293 | 0.306 | 0.097 | 0.268 | 0.225 | 0.087 | 0.091 | 0.100 |
| (S3) *Psudomonas sp*.KF733608 | 0.368 | 0.308 | 1 | 0.700 | 0.643 | 0.775 | 0.605 | 0.500 | 0.385 | 0.718 | 0.388 | 0.447 | 0.476 | 0.089 | 0.523 | 0.391 | 0.111 | 0.114 | 0.171 |
| (S4) *Pseudomonas* sp.KF783212 | 0.421 | 0.293 | 0.700 | 1 | 0.821 | 0.921 | 0.732 | 0.513 | 0.400 | 0.917 | 0.400 | 0.400 | 0.362 | 0.133 | 0.468 | 0.404 | 0.077 | 0.171 | 0.163 |
| (S5) Marine unculture HM363289 | 0.375 | 0.256 | 0.643 | 0.821 | 1 | 0.805 | 0.846 | 0.538 | 0.425 | 0.795 | 0.392 | 0.392 | 0.383 | 0.156 | 0.489 | 0.457 | 0.075 | 0.167 | 0.133 |
| (S6) *Psedomonas* *stultzi* AF094748 | 0.390 | 0.302 | 0.775 | 0.921 | 0.805 | 1 | 0.721 | 0.512 | 0.405 | 0.895 | 0.404 | 0.431 | 0.426 | 0.149 | 0.500 | 0.408 | 0.098 | 0.158 | 0.152 |
| (S7) *Pseudomonas* sp.EU600210 | 0.410 | 0.256 | 0.605 | 0.732 | 0.846 | 0.721 | 1 | 0.538 | 0.462 | 0.707 | 0.420 | 0.420 | 0.383 | 0.156 | 0.556 | 0.489 | 0.075 | 0.135 | 0.133 |
| (S8) *Pseudomonas* sp.KJ911226 | 0.387 | 0.273 | 0.500 | 0.513 | 0.538 | 0.512 | 0.538 | 1 | 0.552 | 0.526 | 0.372 | 0.405 | 0.359 | 0.053 | 0.487 | 0.341 | 0.069 | 0.111 | 0.147 |
| (S9) *Pseudomonas* sp.KJ911227 | 0.379 | 0.258 | 0.385 | 0.400 | 0.425 | 0.405 | 0.462 | 0.552 | 1 | 0.410 | 0.273 | 0.273 | 0.220 | 0.028 | 0.447 | 0.268 | 0.077 | 0.080 | 0.125 |
| (S10) *Pseudomonas stutzi* HM030753 | 0.432 | 0.300 | 0.718 | 0.917 | 0.795 | 0.895 | 0.707 | 0.526 | 0.410 | 1 | 0.380 | 0.438 | 0.400 | 0.111 | 0.447 | 0.383 | 0.108 | 0.176 | 0.140 |
| (S11) *Acidovorax* sp. JQ723680 | 0.256 | 0.262 | 0.388 | 0.400 | 0.392 | 0.404 | 0.420 | 0.372 | 0.273 | 0.380 | 1 | 0.429 | 0.391 | 0.186 | 0.533 | 0.404 | 0.050 | 0.139 | 0.220 |
| (S12) *Frankia* sp.AM040443 | 0.317 | 0.293 | 0.447 | 0.400 | 0.392 | 0.431 | 0.420 | 0.405 | 0.273 | 0.438 | 0.429 | 1 | 0.778 | 0.133 | 0.408 | 0.375 | 0.105 | 0.108 | 0.190 |
| (S13) *Frankia* sp.L40622 | 0.333 | 0.306 | 0.476 | 0.362 | 0.383 | 0.426 | 0.383 | 0.359 | 0.220 | 0.400 | 0.391 | 0.778 | 1 | 0.125 | 0.370 | 0.277 | 0.091 | 0.129 | 0.158 |
| (S14) *Frankia* sp.JN685209 | 0.029 | 0.097 | 0.089 | 0.133 | 0.156 | 0.149 | 0.156 | 0.053 | 0.028 | 0.111 | 0.186 | 0.133 | 0.125 | 1 | 0.087 | 0.146 | 0.045 | 0.048 | 0.148 |
| (S15) *Comamonas* sp.DQ234174 | 0.395 | 0.268 | 0.523 | 0.468 | 0.489 | 0.500 | 0.556 | 0.487 | 0.447 | 0.447 | 0.533 | 0.408 | 0.370 | 0.087 | 1 | 0.383 | 0.079 | 0.081 | 0.195 |
| (S16) *Frankia* sp.HsIi2 | 0.282 | 0.225 | 0.391 | 0.404 | 0.457 | 0.408 | 0.489 | 0.341 | 0.268 | 0.383 | 0.404 | 0.375 | 0.277 | 0.146 | 0.383 | 1 | 0.056 | 0.088 | 0.095 |
| (S17) *Frankia* sp.HsIi8 | 0.040 | 0.087 | 0.111 | 0.077 | 0.075 | 0.098 | 0.075 | 0.069 | 0.077 | 0.108 | 0.050 | 0.105 | 0.091 | 0.045 | 0.079 | 0.056 | 1 | 0.182 | 0.158 |
| (S18) *Frankia* sp.HsIi9 | 0.087 | 0.091 | 0.114 | 0.171 | 0.167 | 0.158 | 0.135 | 0.111 | 0.080 | 0.176 | 0.139 | 0.108 | 0.129 | 0.048 | 0.081 | 0.088 | 0.182 | 1 | 0.105 |
| (S19) *Frankia* sp.HsIi10 | 0.097 | 0.100 | 0.171 | 0.163 | 0.133 | 0.152 | 0.133 | 0.147 | 0.125 | 0.140 | 0.220 | 0.190 | 0.158 | 0.148 | 0.195 | 0.095 | 0.158 | 0.105 | 1 |
|  |  |  |  |  |  |  |  |  |  |  |  |  |  |  |  |  |  |  |  |

Table S1 Proximity matrices of RFLP groups based on Jaccard similarity coefficient
