# Supplementary material for: Deciphering the evolutionary affiliations among bacterial strains (Pseudomonas and Frankia sp.) inhabiting same ecological niche using virtual RFLP and simulation-based approaches
Source: 3 Biotech. 2016 Aug 23;6(2):178. doi: 10.1007/s13205-016-0488-5 (PMC4993716; doi:10.1007/s13205-016-0488-5)
Supplement: Supplementary file 5 — Supplementary material 5 (DOCX 23 kb) [file 13205_2016_488_MOESM5_ESM.docx]

Table S2 Summary of genetic diversity and nucleotide variation in nineteen selected sequences

| Parameters | *Pseudomonas* sp. and  other Proteobacteria | *Pseudomonas* sp. and  Actinobacteria | All strains |
| --- | --- | --- | --- |
| Polymorphic sites (segregating sites, S)  Parsimony informative sites  Nucleotide diversity per site (Pi)  Total number of mutations, (Eta)  Average number of nucleotideide differences, (k)  Average number of nucleotide differences between groups | 283  207  0.43163  423  135.964  198.107 | 86  101  0.45207  120  44.303  58.781 | 86  76  0.46407  160  -  41.766 |
